# Serous Carcinoma of the Endometrium with Mesonephric-Like Differentiation Initially Misdiagnosed as Uterine Mesonephric-Like Adenocarcinoma: A Case Report with Emphasis on the Immunostaining and the Identification of Splice Site *TP53* Mutation

**DOI:** 10.3390/diagnostics11040717

**Published:** 2021-04-18

**Authors:** Sangjoon Choi, Yoon Yang Jung, Hyun-Soo Kim

**Affiliations:** 1 Department of Pathology and Translational Genomics, Samsung Medical Center, Sungkyunkwan University School of Medicine, Seoul 06351, Korea; choisj88@g.skku.edu; 2 Department of Pathology, Myongji Hospital, Hanyang University College of Medicine, Goyang-si 10475, Gyeonggi-do, Korea

**Keywords:** uterus, endometrium, serous carcinoma, mesonephric-like adenocarcinoma, *TP53* mutation

## Abstract

We present herein a rare case of uterine serous carcinoma with mesonephric-like differentiation (SC-MLD) initially misdiagnosed as mesonephric-like adenocarcinoma (MLA). A 51-year-old woman underwent total hysterectomy for a uterine tumor. Histologically, the tumor exhibited various architectures, including papillary, glandular, tubular, cribriform, and cystic. On the basis of this architectural diversity accompanied by intraluminal eosinophilic secretions and intermediate-grade nuclear atypia, the initial diagnosis was MLA. However, the tumor was diffusely and strongly positive for the expression of p16 and negative for the expression of GATA-binding protein 3 (GATA3). Furthermore, we identified a pathogenic tumor protein 53 (*TP53*) mutation affecting an acceptor splice site in intron 9, despite a wild-type p53 immunostaining pattern. The observations of diffuse and strong p16 expression, lack of GATA3 expression, pathogenic *TP53* mutation, and wild-type Kirsten rat sarcoma viral oncogene homolog indicate that this tumor was not an MLA but an SC-MLD. Both uterine SC and MLA can exhibit various histological growth patterns. Our comprehensive clinicopathological and molecular analyses can serve to improve the understanding of this rare condition and help pathologists in making an accurate diagnosis.

## 1. Introduction

Serous carcinoma (SC) of the uterus accounts for only approximately 10% of all endometrial carcinomas [1,2]. However, SC is the most common non-endometrioid endometrial carcinoma and contributes to more than half of recurrence and cancer-related deaths in all endometrial carcinoma cases. When compared with endometrioid adenocarcinoma accompanied by atypical endometrial hyperplasia, SC typically arises in the background of atrophic endometrium or endometrial polyp and is more commonly found in the elderly. SC is characterized by a predilection to spread to the upper abdomen, high risk of recurrence and distant metastasis, and a dismal prognosis [1,3]. Metastatic disease even occurs in cases without myometrial invasion (i.e., minimal uterine SC). Given that most SCs exhibit more advanced stages at the time of presentation and worse survival, extensive surgical staging is recommended by many clinical practice guidelines [4,5].

Mesonephric adenocarcinoma (MA) is a rare but distinct malignancy arising in the uterine cervix and vagina. Cervical MA is typically associated with mesonephric remnant or hyperplasia and is characterized by various growth patterns, including small tubules and glands with intraluminal eosinophilic secretions admixed with papillary, retiform, or solid architecture [6,7,8,9,10,11,12,13]. It also has a unique immunophenotype displaying positive immunoreactivities for GATA-binding protein 3 (GATA3), paired box 2 (PAX2), lack of hormone receptor expression, wild-type p53 immunostaining pattern, and preserved phosphatase and tensin homolog deleted on chromosome 10 (PTEN) immunoreactivity [14,15,16,17,18,19,20]. In addition, most MAs characteristically harbor somatic mutations of Kirsten rat sarcoma 2 viral oncogene homolog (*KRAS*) [8,9,10,11].

Mesonephric-like adenocarcinoma (MLA) of the uterine corpus has been newly included in the fifth edition of the World Health Organization (WHO) classification of female genital tumors [21]. MLA is characterized by significant morphological, immunohistochemical, and genetic similarities to MA, but it has no association with the mesonephric remnant [11,13,22]. Uterine MLA is rare, but it displays an aggressive biological behavior including deeper myometrial invasion, more frequent cervical stromal extension and lymphovascular invasion, more advanced stage, and higher rates of late recurrence and distant metastasis than MA [11,17,22,23]. Furthermore, in a recent study conducted by Euscher et al. [22], the median progression-free and overall survival rates for uterine MLA patients were significantly shorter than those for SC patients.

We recently experienced a rare case of endometrial SC showing mesonephric-like differentiation (SC-MLD). Given that the tumor exhibited various architectural patterns, it was initially misdiagnosed as uterine MLA. Immunostaining and targeted sequencing analysis were performed for accurate determination of the histological subtype. Interestingly, the tumor showed overlapping immunophenotypical features of endometrial SC and uterine MLA, which posed diagnostic challenge. To the best of our knowledge, an endometrial carcinoma with histological and immunophenotypical overlaps between SC and MLA has never been documented in the literature. Notably, our case made us realize that MLA should not be diagnosed solely on the basis of histological features. Ancillary tests including immunostaining and molecular analysis are essential for the differential diagnosis. Since the clinical course and outcome are different between SC and MLA patients, it is critical for pathologists to accurately determine the tumor origin and histological subtype for appropriate pathological staging and treatment planning. In this report, we aimed to provide a thorough clinicopathological description of SC-MLD, as well as its immunophenotype and genetic features.

## 2. Case Presentation

A 51-year-old woman diagnosed with advanced-stage endometrial carcinoma was referred to our institution for the postoperative treatment of multiple metastases to the peritoneum, lymph nodes, lungs, and bone. She had undergone total hysterectomy with right salpingo-oophorectomy at another hospital. Postoperative abdominopelvic magnetic resonance imaging revealed multiple seeding masses in the abdominal and pelvic peritoneum, multiple enlarged lymph nodes in the bilateral iliac chains and retroperitoneum, and a suspected metastatic lesion in the left posterior iliac bone. Chest computed tomography (CT) revealed innumerable, well-defined enhancing nodules throughout the bilateral lungs and enlarged lymph nodes in the right hilar, interlobar, and anterior cardiophrenic areas, all of which were suspected to be metastatic lesions. Positron emission tomography/CT (PET/CT) revealed multiple hypermetabolic lymph nodes in the right mediastinal, right cardiophrenic, retroperitoneal, and bilateral iliac areas, all of which were suggestive of metastatic lymphadenopathy. Foci of increased fluorodeoxyglucose uptake were also noted in the bilateral lungs, omentum, and left posterior iliac bone. We examined copies of the outside medical records and pathological report enclosed with the hematoxylin and eosin-stained slides. We also performed immunostaining and targeted sequencing using the unstained slides from the previous hospital.

According to the medical records, the uterus measured 13.0 × 7.0 × 6.0 cm. Its contour was distorted by extensive myometrial involvement of the tumor. The anterior low uterine segment was almost entirely involved by the tumor, and the uterine serosa facing the bladder was fibrotic. The myometrium had a 4.0-cm relatively well-circumscribed, gray-to-white, solid mass involving the anterior uterine wall and protruding into the endometrial cavity. The right ovary had a 0.8-cm white, solid mass, suspected to be a metastatic lesion. The right salpinx measured 7.0 × 1.5 × 1.5 cm and showed luminal dilatation.

**Figure 1:**
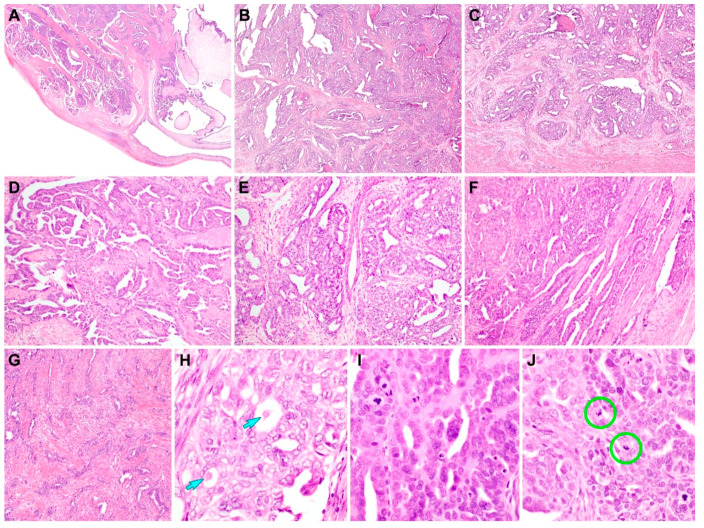
Histological features observed on initial microscopic examination. (**A**) Scanning magnification revealing that the tumor involved the entire myometrial thickness. The irregular glands and cysts contained papillary cellular buds. We considered that the presence of large, cystically dilated spaces was a very uncommon finding if this tumor is endometrial endometrioid adenocarcinoma or serous carcinoma. (**B**) Glandular (left half) and solid (right half) tumor growth patterns resembling grade 2 endometrioid adenocarcinoma of the endometrium. (**C**) At the invasive front, the infiltrating tumor glands accompanied by strong desmoplastic stromal reaction. (**D**) Intraluminal floating papillary tufts and micropapillae morphologically similar to those of serous carcinoma. (**E**) Small tubular and glandular patterns merged into each other and forming a cribriform architecture. The tubules are small, round, uniform, and closely packed; lined with cuboidal-to-low columnar epithelial cells; and accompanied by little intervening stroma, producing a back-to-back appearance. (**F**) In some foci, elongated, slit-like branching tubules lined by flattened epithelial cells and containing papillary tufts (right half; retiform pattern). (**G**) In areas of poorly differentiated carcinoma, the tumor cells with poorly formed, angulated glands, cords, and small nests. (**H**) The nuclei of tumor cells exhibiting tubular and glandular growth patterns show intermediate-grade nuclear atypia, similar to that observed in grade 1 or 2 endometrioid adenocarcinoma. The intraluminal eosinophilic secretions (blue arrows) occasionally observed in tubules and glands. (**I**) Tumor cells growing in solid and papillary patterns displaying moderate-to-severe nuclear pleomorphism and frequent mitotic figures (left upper quadrant) and apoptotic bodies (right lower quadrant). (**J**) Atypical mitotic figures (green circles) are often present. On the basis of the architectural diversity and intraluminal eosinophilic secretions, we initially considered this tumor as mesonephric-like adenocarcinoma. Staining method: **A**–**J**, hematoxylin and eosin staining. Original magnification: (**A**) ×10; (**B**,**C**) ×40; (**D**–**G**) ×100; (**H**–**J**) ×400.

Histologically, the tumor had an ill-defined border and involved the entire myometrial thickness. The right ovary showed a metastatic carcinoma, whose histological features were identical to those of the primary lesion. The uterine tumor showed several large, cystically dilated spaces (Figure 1A). Many variable-sized papillary fronds with delicate fibrovascular cores or densely hyalinized stroma occupied the cystic spaces. Most papillae exhibited a hierarchical growth pattern, but in some areas, long, slender micropapillae directly protruded from the surfaces of large, edematous papillae. In addition to the papillary architecture, the tumor consisted of compactly aggregated small tubules and glands (Figure 1B). These tubules and endometrioid-like glands were lined by cuboidal and columnar epithelial cells, respectively. They were anastomosed and fused with each other, resulting in a solid and cribriform architecture. Most tumor cell nuclei displayed intermediate-grade atypia characterized by mild hyperchromasia, moderate enlargement, mild-to-moderate pleomorphism, occasional small nucleoli, and rare mitotic figures. At the invasive front, the cribriform glands were associated with prominent stromal desmoplasia (Figure 1C). Representative photomicrographs of diverse growth patterns are shown in Figure 1D (papillary pattern), Figure 1E (small tubular, glandular, and cribriform patterns), and Figure 1F (retiform pattern). As minor patterns, elongated glands with poorly formed lumina haphazardly invaded the myometrium and were accompanied by a fibromyxoid stromal reaction (Figure 1G). Some of them were devoid of lumen formation and appeared as angulated cords and small nests of tumor cells. Intraluminal eosinophilic secretions were often noted in small tubules and endometrioid-like glands as well as cribriform glands (Figure 1H). In addition, some scattered foci demonstrated high-grade nuclear atypia characterized by enlargement, severe pleomorphism, conspicuous nucleoli, and brisk mitotic activity (Figure 1I), as well as occasional atypical mitotic figures (Figure 1J). On the basis of our observations of architectural diversity, intermediate-grade nuclear atypia, and intraluminal eosinophilic secretions, we first considered the possibility of MLA. Even though the presence of cystically dilated spaces and focal but obvious high-grade nuclear atypia were not compatible with typical MLA, we previously found that those unusual features can be identified in a small subset of uterine MLA [11].

**Figure 2:**
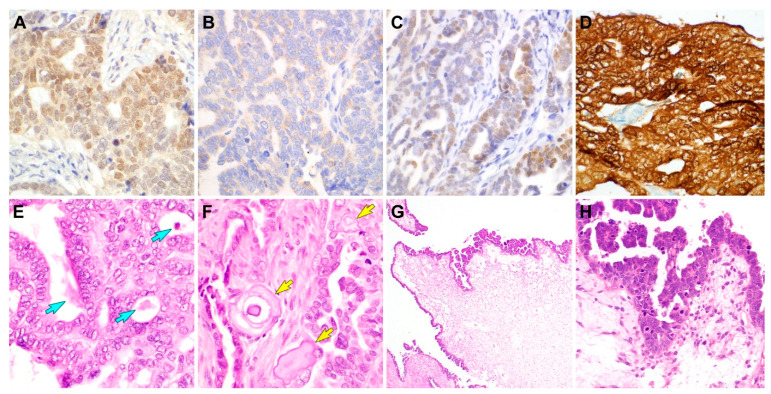
Immunostaining results (**A**–**D**) and histological features detected on re-examination (**E**–**H**). (**A**) Paired box 2 expression observed in most tumor cell nuclei, though the staining intensity was weak-to-moderate. (**B**) GATA-binding protein 3 expression was absent, which does not support uterine mesonephric-like adenocarcinoma (MLA). (**C**) p53 expression was patchy, with faint-to-weak staining intensity in approximately half of the tumor cell nuclei. This wild-type p53 immunostaining pattern is compatible with MLA but not with endometrial serous carcinoma (SC). (**D**) Uniform and intense nuclear and cytoplasmic p16 immunoreactivity in the entire tumor tissue strongly indicated the possibility of SC. These immunostaining results made us re-examine the hematoxylin and eosin-stained slides. (**E**) We found a few additional microscopic foci showing intraluminal eosinophilic secretions (blue arrows). (**F**) The presence of psammomatous microcalcifications (yellow arrows), which was not identified in initial microscopic examination, further supported SC. (**G**) Large papillae with edematous stroma possessing many variable-height micropapillae along their surface. (**H**) High-power magnification of the micropapillae revealing nuclear hyperchromasia and severe pleomorphism as well as numerous mitotic figures and many apoptotic bodies, all of which are compatible with SC. Staining method: (**A**–**D**) polymer method; (**E**–**H**) hematoxylin and eosin staining. Original magnification: (**A**–**F**) ×400; (**G**) ×40; (**H**) ×200.

Immunostaining was performed using a compact polymer method (Bond Polymer Refine Detection kit, Leica Biosystems, Newcastle, United Kingdom) [13,24,25,26,27,28,29,30,31,32,33]. The tumor cells showed focal nuclear PAX2 immunoreactivity with mild-to-moderate staining intensity (Figure 2A). Nuclear GATA3 did not show immunoreactivity, even though it was expressed in the cytoplasm of some tumor cells (Figure 2B). p53 immunostaining revealed that approximately half of the tumor cells reacted with the antibody patchily with weak-to-moderate staining intensity, indicating wild-type p53 immunostaining pattern (Figure 2C). The tumor cells demonstrated diffuse and strong p16 immunoreactivity in both the nuclei and cytoplasm (Figure 2D). The estrogen receptor (ER) and progesterone receptor (PR) statuses were negative. Collectively, the lack of hormone receptor expression and PAX2 expression in the well-formed tubules and glands favored MLA but GATA3 negativity did not. Concurrently, the p16 expression pattern favored SC, but wild-type p53 immunostaining pattern was not compatible with SC. The majority of uterine SC typically expresses p16 uniformly and strongly [34]; further, we previously noted that none of the MLA cases exhibited diffuse and strong p16 expression [11]. Thus, we considered that the immunostaining results are not likely indicative of MLA but of SC-MLD. During the initial microscopic examination, the architectural diversity and small tubules with intraluminal eosinophilic secretions were thought to be strongly indicative of MLA. However, there were foci showing severe nuclear pleomorphism and brisk mitotic activity; thus, we did not rule out SC in the differential diagnosis. Given that gynecological tumors harboring unusual tumor protein 53 (*TP53*) mutation can present with a wild-type p53 immunostaining pattern [35], we further conducted targeted sequencing analysis.

Targeted sequencing was performed to analyze single-nucleotide variations, insertions, and deletions and copy number variations using CancerSCAN (Samsung Genome Institute, Samsung Medical Center, Seoul, Republic of Korea) [36]. We did not identify any pathogenic mutation in well-known hot spots of *TP53* exons. Instead, interestingly, we noted that the tumor harbored a *TP53* mutation in the intron inclusion between exons 9 and 10 (NM_000546.5(*TP53*):c.994-1G>C). We verified this unusual *TP53* mutation affecting an acceptor splice site in intron 9 to be pathogenic in two disease-related databases, ClinVar (National Center for Biotechnology Information, United States National Library of Medicine, Bethesda, Maryland, United States of America) and Catalogue of Somatic Mutations in Cancer (Wellcome Sanger Institute, Hinxton, Cambridgeshire, United Kingdom). The identification of this unusual but definitely pathogenic mutation confirmed the wild-type p53 immunostaining pattern and the diagnosis of SC. We thoroughly re-examined all available hematoxylin and eosin-stained slides and confirmed diverse architectural patterns. In particular, the tumor tissue showed two dominant patterns, papillary and small tubular/glandular patterns, in almost equal proportions. The intraluminal eosinophilic secretions were still identified relatively easily (Figure 2E). However, in a few microscopic foci, we detected several round, concentrically laminated calcospherites that were located closely adjacent to the glands and morphologically compatible with psammomatous microcalcifications (Figure 2F). Furthermore, we found several microscopic areas of variable-sized papillae and micropapillae overlying large, edematous stroma (Figure 2G), showing severe nuclear pleomorphism and brisk mitotic activity (Figure 2H), which were overlooked during the initial microscopic examination. We acknowledged that we missed the presence of high-grade nuclear atypia not only in floating papillary tufts and intraluminal micropapillary projections but also in tubules and glands.

**Figure 3:**
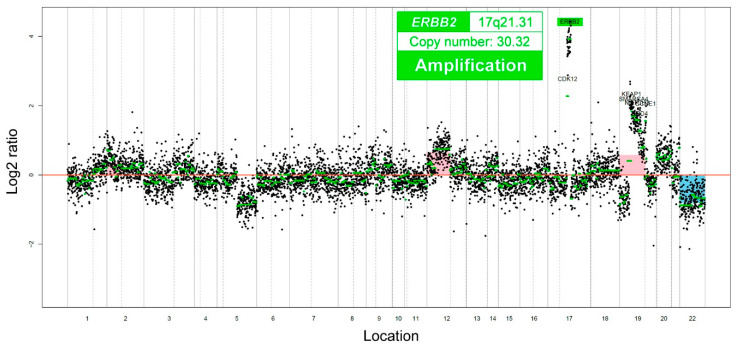
Copy number plots. The Erb-b2 receptor tyrosine kinase 2 (*ERBB2*) gene is markedly amplified.

CancerSCAN (Samsung Genome Institute) also revealed the amplification of the Erb-b2 receptor tyrosine kinase (*ERBB2*) gene, gains of chromosomal 12p and 19p, and loss of 22q (Figure 3). In contrast, the pathogenic *KRAS* mutation, characteristic of MLA, was not identified. No pathogenic mutation was detected in PTEN, AT-rich interactive domain 1A, β-catenin, and phosphatidylinositol-4,5-bisphosphate 3-kinase catalytic subunit alpha, which are known to be mutated in endometrioid adenocarcinoma. Finally, we decided that this case should be diagnosed as SC showing morphological and immunophenotypical features closely resembling MLA. The following observations designated this tumor not as MLA but as SC-MLD: diffuse and strong p16 expression, pathogenic *TP53* mutation, lack of GATA3 expression, and wild-type *KRAS*.

Final pathological diagnosis of SC-MLD with multiple metastases to the following organs: right adnexa; abdominopelvic peritoneum; pelvic, para-aortic, cardiophrenic, and mediastinal lymph nodes; lungs; and iliac bone was made. The International Federation Gynecology and Obstetrics stage was designated as IVB. She received postoperative chemotherapy with taxane and platinum-based combination regimen. After six cycles of combination chemotherapy, significant decreases in the size and number of metastatic lesions were noted in imaging studies. Follow-up PET/CT images revealed significantly decreased fluorodeoxyglucose uptake in the metastatic masses of the lungs, peritoneum, lymph nodes, and bone. However, CT images showed several residual tumors in the left external iliac region (greatest dimension, 0.8 cm) and omentum (greatest dimension, 4.6 cm). She underwent left salpingo-oophorectomy with bilateral pelvic lymph node dissection, omentectomy, and appendectomy. Microscopic examination confirmed that the left adnexa and omentum were involved by the metastatic tumors, which exhibited the same immunoprofile as that of the primary uterine tumor. She was scheduled to receive an additional three cycles of chemotherapy.

## 3. Discussion

Uterine SC is a clinically aggressive, high-grade carcinoma [37]. Complex papillary and glandular growth patterns with high-grade cytomorphology are essential diagnostic criteria for SC [21]. Nearly every SC harbors pathogenic *TP53* mutation and displays mutant p53 immunostaining pattern [38]. Notably, SC invariably shows uniform and intense p16 expression, which serves as a diagnostic marker along with p53 [34]. Uterine MLA is an unusual subset of malignancies arising in the uterine corpus, representing less than 1% of uterine carcinomas [7]. Despite its low incidence, MLA has been reported to exhibit aggressive behavior [22]. It is histologically characterized by diverse architectural patterns including tubular, glandular, papillary, solid, retiform, glomeruloid, and comedonecrosis-like patterns [11]. Given that both MLA and SC exhibit papillary and glandular architecture with high-grade nuclear atypia, they should be included in the differential diagnosis of uterine tumors exhibiting various growth patterns. Immunostaining results that are specific to each type are usually sufficient for the differential diagnosis. For example, positive immunoreactivities for GATA3 and PAX2, which are frequently observed in MLA [11,16,18,19], are rarely identified in SC. In contrast, an aberrant p53 immunostaining pattern and diffuse and strong p16 immunoreactivity are extremely rare in MLA and support the diagnosis of SC. In the current case, various growth patterns other than papillae and glands (small tubule formation, cribriforming, and retiform tubules) and relatively easily identifiable intraluminal eosinophilic secretions supported MLA because SC usually consists predominantly of papillary and glandular structures. In contrast, the presence of severe nuclear pleomorphism, brisk mitotic activity, and psammomatous microcalcifications favored SC. Furthermore, immunostaining results also shared the features of MLA and SC. The tumor cells were positive for PAX2 but negative for GATA3. Diffuse and strong nuclear and cytoplasmic p16 immunoreactivity supported the diagnosis of SC, but the p53 immunostaining pattern was wild-type, which is a very uncommon finding in SC. Despite the complicated histological and immunohistochemical features, we finally confirmed the diagnosis of SC-MLD using targeted sequencing analysis, which revealed that the tumor harbored a splice site *TP53* mutation but not a *KRAS* mutation. The histological, immunohistochemical, and molecular characteristics of our case are summarized in Table 1.

We thoroughly re-examined the slides and found some histological features that were indicative of SC rather than MLA. The intraluminal eosinophilic secretions within the tubules and glands were not as dense as those typically observed in mesonephric hyperplasia or a remnant. Variable-sized papillae with edematous stroma, micropapillae exhibiting severely pleomorphic nuclei and markedly increased mitotic activity with atypical mitotic figures, and scattered psammoma bodies were compatible with SC. One of the characteristic morphological features of MLA is the presence of eosinophilic secretions within the tubular and glandular lumina [11,22]. Compared with SC, MLA has deeply eosinophilic, hyaline-, or colloid-like secretions, which are typically bright pink on hematoxylin and eosin staining and similar to those observed in benign mesonephric lesions or thyroid follicles [11,22]. Moreover, the intraluminal secretions of MLA usually conform to the contours of the glands in which they are found. In contrast, SC has an irregular, shattered intraglandular materials that are not the same shape of the lumen. However, because the nature of intraluminal secretions is neither specific nor pathognomonic for any of the two types, ancillary tests including immunostaining and targeted sequencing analysis are recommended for an accurate diagnosis.

PAX2 is known as a highly sensitive marker for benign and malignant mesonephric lesions, and all previously reported MLA cases showed diffuse nuclear staining [11,18]. However, we found that approximately 56% of uterine SCs also express PAX2 [39], indicating that MLA cannot be distinguished from SC solely on the basis of positive PAX2 immunoreactivity. Furthermore, PAX2 immunostaining result can be a potential diagnostic pitfall in terms of the staining intensity. We recently demonstrated that non-neoplastic endometrial glandular epithelial cells displayed weak-to-moderate nuclear PAX2 immunoreactivity, whereas MLA showed uniform and strong staining intensity in all cases examined [11]. The current case exhibited weak-to-moderate PAX2 expression, which did not strongly support the diagnosis of MLA. Use of a panel of markers such as GATA3 and CD10 and awareness of the lack of specificity of PAX2 will help avoid misdiagnosis, particularly when MLA and other uterine carcinomas showing mesonephric-like differentiation are suspected.

We observed an unusual *TP53* mutation affecting splice site of intron 9. While this mutation has been documented in patients with Li–Fraumeni syndrome [40,41], to the best of our knowledge, this has not been previously reported in endometrial SC. Schultheis et al. [38] reported that splice site *TP53* mutations are relatively uncommon in endometrial SC. Though the effects of these mutations have not been well characterized owing to the limited number of cases, it is reasonable to assume that, like other known point mutations, insertions, and deletions affecting *TP53* exons, this rare pathogenic mutation affecting the splice site may also be involved in the pathogenesis and development of SC in the current case. In addition, we previously demonstrated that the splice site *TP53* mutation can result in a wild-type p53 immunostaining pattern in tubo-ovarian or peritoneal high-grade SC [35]. The discrepancy between p53 immunostaining result and *TP53* mutational status observed in this case is consistent with our previous observation. When a high-grade endometrial carcinoma with serous histology exhibits a wild-type p53 immunostaining pattern, molecular tests such as targeted sequencing analysis or Sanger sequencing should be performed to confirm the presence of uncommon but pathogenic *TP53* mutation [35].

We detected not only an unusual *TP53* mutation but also ERBB2 amplification using targeted sequencing. *ERBB2* amplification is observed in approximately 30% of endometrial SC cases [42]. As trastuzumab can significantly improve the prognosis of patients with uterine SC showing human epidermal growth factor receptor 2 overexpression or *ERBB2* gene amplification [43], the identification of *ERBB2* amplification can provide additional therapeutic options. In the era of precision oncology, targeted sequencing helps pathologists make an accurate diagnosis and identify the cancer mutation for targeted therapy.

## 4. Conclusions

In summary, we have presented of a case of endometrial SC-MLD initially misdiagnosed as MLA. Though diverse architectural patterns including small tubular, glandular, and papillary patterns can be observed in both uterine SC and MLA, the severe nuclear pleomorphism, brisk mitotic activity, and presence of psammomatous microcalcifications in the case supported SC. Lack of GATA-3 expression, diffuse and strong nuclear and cytoplasmic p16 immunoreactivity, and the presence of pathogenic *TP53* mutation designated this tumor as SC-MLD rather than MLA. Weak-to-moderate PAX2 immunoreactivity and wild-type p53 immunostaining pattern could be diagnostic pitfalls, and splice site *TP53* mutation caused the discordant p53 results between immunostaining and targeted sequencing. For cases with high-grade endometrial carcinoma showing wild-type p53 immunostaining pattern, targeted sequencing can help pathologists confirm the presence of *TP53* mutation. Our comprehensive clinicopathological and molecular analyses can serve to improve the understanding of this rare condition and help pathologists in making an accurate diagnosis.

## Figures and Tables

**Table 1:** Summary of histological features, immunostaining results, and targeted sequencing results.

Parameter		Result	Interpretation
Histology	Architectural diversity	Present	Supports both MLA and SC
	Papillary and solid patterns	Present	Support both MLA and SC
	Small tubular and glandular patterns	Present	Support MLA; possible in SC
	Intraluminal eosinophilic secretions	Present	Support MLA
	Psammomatous microcalcifications	Present	Support SC
	Severe nuclear pleomorphism	Present	Support SC; possible in MLA
	Brisk mitotic activity	Present	Support SC; possible in MLA
Immunostaining	ER	Negative	Support both MLA and SC
	PR	Negative	Support both MLA and SC
	PTEN	Preserved	Support both MLA and SC
	PAX2	Diffusely positive with weak-to-moderate intensity	Support MLA
	GATA3	Negative	Do not support MLA
	p16	Diffusely positive with strong intensity	Support SC
	p53	Wild-type pattern	Do not support SC
Targeted sequencing	*KRAS*	Wild type	Do not support MLA
	*TP53*	Pathogenic mutation (splice acceptor variant)	Support SC
	*ERBB2*	Amplification	Support SC; unknown in MLA

ER, estrogen receptor; PR, progesterone receptor; PTEN, phosphatase and tensin homolog deleted on chromosome 10; PAX2, paired box 2; GATA3, GATA-binding protein 3; *KRAS*, Kirsten rat sarcoma viral oncogene homolog; *TP53*, tumor protein 53; *ERBB2*, erb-b2 receptor tyrosine kinase 2.
